# EXPERIENCE OF PHARMACOGENETIC TESTING IN THE TREATMENT OF ANTIPSYCHOTICS

**DOI:** 10.1192/j.eurpsy.2023.2272

**Published:** 2023-07-19

**Authors:** M. Kaydan, N. Zakharova

**Affiliations:** Psychiatric Hospital no. 1 Named after N.A. Alexeev of the Department of Health of Moscow, Moscow, Russian Federation

## Abstract

**Introduction:**

One of the promising methods for optimizing treatment in order to achieve high-quality remissions is a personalized approach to prescribing therapy in the form of pharmacogenetic testing, the feasibility of which has already been substantiated and proven in a number of clinical guidelines. By the beginning of 2022, several influential regulatory and expert organizations recommend considering the results of genetic testing when prescribing therapy. Thus, personalization of antipsychotic therapy is being introduced in the world practice.

**Objectives:**

To establish the significance of pharmacogenetic markers that determine the efficacy and safety of antipsychotic therapy in patients with schizophrenia in clinical practice.

**Methods:**

The study included 264 patients (141 men, 123 women; 27.3 ± 4.5 years) from among the first hospitalized in a psychiatric hospital in the period 2018-2020, meeting the inclusion criteria (psychosis within the schizophrenia spectrum disorders; consent to participate in research). Non-inclusion criteria - signs of organic brain damage; alcohol or substance abuse; somatic pathology in the stage of decompensation). The examination took place in three stages - in the first days of hospitalization at the peak of the acute condition and during the formation of remission - after 6 and 12 months. Genetic analysis was performed using high-density biochips from Illumina CoreExome Bead (Illumina Inc, USA). During the follow-up observation, some patients dropped out due to refusal to undergo examination, change of diagnosis or change of place of residence. After 6 and 12 months, it was possible to trace the dynamics of the state of 91 patients (50 men, 41 women; 24.9 ± 4.6 years).

**Results:**

Based on the follow-up results, two types of schizophrenia dynamics were identified - with a relatively favorable and unfavorable course. The formation of a relatively stable remission corresponding to the criteria proposed by the working group was noted in 47 patients (51.6%), carriers of gene polymorphisms: DRD2 rs1799732 (del); COMT rs4680(GG); BDNF rs6265 (CC); ANKK1 rs1800497 (GG); MC4R rs489693 (AA); ABCB1 rs1045642 and ABCC1 rs212090 (GG). An unfavorable course with the ineffectiveness of antipsychotics was found in 48.4% of cases in patients with DRD2 rs1799732 (G/del) carriers; COMT rs4680(AA); BDNF rs6265 (TT); ANKK1 rs1800497 (AA); MC4R rs489693 (GG); ABCB1 rs1045642 and ABCC1 rs212090 (AA).

**Conclusions:**

After analyzing the results of genetic testing and clinical and dynamic characteristics of the course of schizophrenia, we can talk about the relationship between the establishment of high-quality remission in the presence of polymorphisms in the genotype, whose role has been proven in terms of the effectiveness and safety of antipsychotics.

**Disclosure of Interest:**

None Declared

